# Alkaloids Isolated from Natural Herbs as the Anticancer Agents

**DOI:** 10.1155/2012/485042

**Published:** 2012-09-04

**Authors:** Jin-Jian Lu, Jiao-Lin Bao, Xiu-Ping Chen, Min Huang, Yi-Tao Wang

**Affiliations:** State Key Laboratory of Quality Research in Chinese Medicine (University of Macau) and Institute of Chinese Medical Sciences, University of Macau, Avenue Padre Toma's Pereira, Taipa 999078, Macao, China

## Abstract

Alkaloids are important chemical compounds that serve as a rich reservoir for drug discovery. Several alkaloids isolated from natural herbs exhibit antiproliferation and antimetastasis effects on various types of cancers both *in vitro* and *in vivo*. Alkaloids, such as camptothecin and vinblastine, have already been successfully developed into anticancer drugs. This paper focuses on the naturally derived alkaloids with prospective anticancer properties, such as berberine, evodiamine, matrine, piperine, sanguinarine, and tetrandrine, and summarizes the mechanisms of action of these compounds. Based on the information in the literature that is summarized in this paper, the use of alkaloids as anticancer agents is very promising, but more research and clinical trials are necessary before final recommendations on specific alkaloids can be made.

## 1. Introduction

Alkaloids are a highly diverse group of compounds that contain a ring structure and a nitrogen atom. In most cases, the nitrogen atom is located inside the heterocyclic ring structure [1]. A classification based on biosynthetic pathways is mostly used to categorize different alkaloid [1]. Alkaloids have a wide distribution in the plant kingdom and mainly exist in higher plants, such as those belonging to Ranunculaceae, Leguminosae, Papaveraceae, Menispermaceae, and Loganiaceae [1]. Moreover, several alkaloids exhibit significant biological activities, such as the relieving action of ephedrine for asthma, the analgesic action of morphine, and the anticancer effects of vinblastine [1–4]. In fact, alkaloids are among the most important active components in natural herbs, and some of these compounds have already been successfully developed into chemotherapeutic drugs, such as camptothecin (CPT), a famous topoisomerase I (TopI) inhibitor [5], and vinblastine, which interacts with tubulin [4].

**Figure 1:**
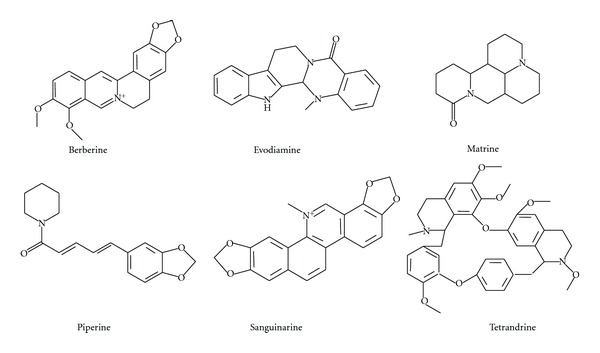
The chemical structures of berberine, evodiamine, matrine, piperine, sanguinarine, and tetrandrine.

Herein, we searched the PubMed database and the naturally derived alkaloids, such as berberine, evodiamine, matrine, piperine, sanguinarine, and tetrandrine (Figure 1), which have relatively more anticancer studies, have been selected for reviewing. Other alkaloids (such as chelerythrine, chelidonine, fagaronine, lycorine, nitidine chloride, and solanine) lacking systematic anticancer investigations have also been mentioned. The aim of this paper is to summarize and investigate the mechanisms of action of these compounds to accelerate the discovery of anticancer drugs derived from alkaloids. We propose that the development of alkaloids into new anticancer agents has a bright future despite some difficulties.

## 2. Alkaloids with Anticancer Effects and the**  **Related Mechanisms

### 2.1. Berberine

Berberine (Figure 1) is an isoquinoline alkaloid widely distributed in natural herbs, including *Rhizoma Coptidis*, a widely prescribed Chinese herb [6]. It has a broad range of bioactivities, such as antiinflammatory, antibacterial, antidiabetes, antiulcer, sedation, protection of myocardial ischemia-reperfusion injury, expansion of blood vessels, inhibition of platelet aggregation, hepatoprotective, and neuroprotective effects [7–11]. Berberine has been used in the treatment of diarrhea, neurasthenia, arrhythmia, diabetes, and so forth [11]. Several studies have shown that berberine has anticancer potentials by interfering with the multiple aspects of tumorigenesis and tumor progression in both *in vitro* and *in vivo* experiments. These observations have been well summarized in the recent reports [12–14]. Berberine inhibits the proliferation of multiple cancer cell lines by inducing cell cycle arrest at the G_1_ or G_2_/M phases and by apoptosis [12, 15, 16]. In addition, berberine induces endoplasmic reticulum stress [15] and autophagy [17] in cancer cells. However, compared with clinically prescribed anticancer drugs, the cytotoxic potency of berberine is much lower, with an IC_50_ generally at 10 *μ*M to 100 *μ*M depending on the cell type and treatment duration *in vitro *[12]. Besides, berberine also induces morphologic differentiation in human teratocarcinoma cells [18]. Inhibition of tumor invasion and metastasis is an important aspect of berberine's anticancer activities [19, 20]. A few studies have reported berberine's inhibition of tumor angiogenesis [21, 22]. In addition, its combination with chemotherapeutic drugs or irradiation could enhance the therapeutic effects [23, 24]. Recently, a study reported that berberine also showed promising chemopreventive efficacy in hamster buccal pouch carcinogenesis [25].

The potential molecular targets and mechanisms of berberine are rather complicated. Berberine interacts with DNA or RNA to form a berberine-DNA or a berberine-RNA complex, respectively [26, 27]. Berberine is also identified as an inhibitor of several enzymes, such as N-acetyltransferase (NAT), cyclooxygenase-2 (COX-2), and telomerase [12]. Other mechanisms of berberine are mainly related to its effect on cell cycle arrest and apoptosis, including regulation of cyclin-dependent kinase (CDK) family of proteins [12, 28] and expression regulation of B-cell lymphoma 2 (Bcl-2) family of proteins (such as Bax, Bcl-2, and Bcl-xL) [12, 15, 28], and caspases [15, 28]. Furthermore, berberine inhibits the activation of the nuclear factor *κ*-light-chain-enhancer of activated B cells (NF-*κ*B) and induces the formation of intracellular reactive oxygen species (ROS) in cancer cells [12, 15]. Interestingly, these effects might be specific for cancer cells [12]. The effect of berberine on invasion, migration, metastasis, and angiogenesis is mediated through the inhibition of focal adhesion kinase (FAK), NF-*κ*B, urokinase-type plasminogen-activator (u-PA), matrix metalloproteinase 2 (MMP-2), and matrix metalloproteinase 9 (MMP-9) [20, 29]; reduction of Rho kinase-mediated Ezrin phosphorylation [19]; reduction of the expression of COX-2, prostaglandin E, and prostaglandin E receptors [30]; downregulation of hypoxia-inducible factor 1 (HIF-1), vascular endothelial growth factor (VEGF), proinflammatory mediators [21, 22], and so forth. 

### 2.2. Evodiamine

Evodiamine (Figure 1), a quinolone alkaloid, is one of the major bioactive compounds isolated from the Chinese herb *Evodia rutaecarpa*. It possesses antianxiety, antiobese, antinociceptive, antiinflammatory, antiallergic, and anticancer effects. Besides, it has thermoregulation, protection of myocardial ischemia-reperfusion injury and vessel-relaxing activities [11, 31–34]. Evodiamine exhibits anticancer activities both *in vitro* and *in vivo* by inducing the cell cycle arrest or apoptosis, inhibiting the angiogenesis, invasion, and metastasis in a variety of cancer cell lines [35–39]. It presents anticancer potentials at micromolar concentrations and even at the nanomolar level in some cell lines *in vitro* [40, 41]. Evodiamine also stimulates autophagy, which serves as a survival function [42]. Compared with other compounds, evodiamine is less toxic to normal human cells, such as human peripheral blood mononuclear cells [37, 43]. It also inhibits the proliferation of adriamycin-resistant human breast cancer NCI/ADR-RES cells both *in vitro* and in Balb-c/nude mice [44]. Evodiamine (10 mg/kg) administrated orally twice daily significantly inhibits the tumor growth [44]. Moreover, treatment with 10 mg/kg evodiamine from the 6th day after tumor inoculation into mice reduces lung metastasis and does not affect the body weight of mice during the experimental period [35].

Evodiamine inhibits TopI enzyme, forms the DNA covalent complex with a similar concentration to that of CPT, and induces DNA damage [45–47]. However, TopI may not be the main target of this compound. Cancer cells treated with evodiamine exhibit G_2_/M phase arrest [44, 48, 49] rather than S phase arrest, which is not consistent with the mechanism of classic TopI inhibitors, such as CPT. Therefore, other targets aside from TopI may also be important for realizing the anticancer potentials of evodiamine. This statement is supported by the fact that evodiamine has effect on tubulin polymerization [49]. Exposure to evodiamine rapidly increases intracellular ROS followed by an onset of mitochondrial depolarization [50]. The generation of ROS and nitric oxide acts in synergy and triggers mitochondria-dependent apoptosis [42]. Evodiamine also induces caspase-dependent and caspase-independent apoptosis, downregulates Bcl-2 expression, and upregulates Bax expression in some cancer cells [38, 40]. The phosphatidylinositol 3-kinase/Akt/caspase and Fas ligand (Fas-L)/NF-*κ*B signaling pathways might account for evodiamine-induced cell death. Moreover, these signals could be increased by the ubiquitin-proteasome pathway [41].

### 2.3. Matrine

Matrine (Figure 1) is a major alkaloid found in many *Sophora* plants, including *Sophora flavescens *Ait. [51]. It exhibits a wide range of pharmacological properties such as antibacterial, antiviral, antiinflammatory, antiasthmatic, antiarrhythmic, antiobesity, anticancer, diuretic, choleretic, hepatoprotective, nephroprotective, and cardioprotective effects [11, 52–58]. It has been used for treatment of bacillary dysentery, enteritis, malignant pleural effusion, and so forth in China [11], and the anticancer effects have also been widely studied [59–61]. Although the needed concentration of matrine to inhibit cancer cell proliferation is relatively high (i.e., at millimolar level) [59, 60], it has no significant effects on the viability of normal cells [60]. Matrine inhibits the proliferation of various types of cancer cells mainly through mediation of G_1_ cell cycle arrest or apoptosis [59, 60, 62–64]. Apoptosis and autophagy could be both induced by matrine in human cancer cells, such as hepatoma G2 cells and SGC-7901 cells [65, 66]. Matrine also induces the differentiation of K562 cells and presents antiangiogenesis activities [67, 68]. The *in vivo* anticancer efficacy of matrine has already been evaluated in H22 cells, MNNG/HOS cells, 4T1 cells and BxPC-3 cells in BALB/c mice, among others [60, 61, 68, 69]. For example, matrine at 50 mg/kg or 100 mg/kg inhibits MNNG/HOS xenograft growth [61], and it reduces the pancreatic tumor volumes compared to those of control at the similar doses [60].

However, the exact targets of matrine are still unclear. Matrine affects many proteins involved in cell proliferation or apoptosis, such as E2F-1, Bax, Bcl-2, Fas, and Fas-L [59–61, 63, 64, 70]. It inhibits cancer cell invasion partially through inhibition of MMP-2 and MMP-9 expression and modulation of the NF-*κ*B signaling pathway [71–73]. Matrine has been used in China for cancer therapy. The direct inhibition of cancer proliferation by this compound seems not to be the exact mechanism that could explain the reason for its application in cancer treatment.

### 2.4. Piperine

Piperine (Figure 1), a piperidine alkaloid isolated from *Piper nigrum *and* Piper longum*, is a compound found in famous spices that have been used for centuries [74]. It exhibits antioxidant, antiinflammatory, antidiarrheal, anticonvulsant, antimutagenic, hypolipidemic, promoting bile secretion, and tumor inhibitory activities [11, 75, 76]. It is also a known antidepressant of the central nervous system [77, 78]. The chemopreventive effects of piperine against several kinds of carcinogen, such as benzo(a)pyrene, and 7,12-dimethyl benz(a)anthracene, show its potential as a cancer preventive agent [79–85]. Administration of piperine (50 mg/kg or 100 mg/kg per day for 7 days) inhibits solid tumor development in mice transplanted with sarcoma 180 cells [86]. A recent study has shown that piperine inhibits breast stem cell self-renewal and does not cause toxicity to differentiated cells [87]. It has been demonstrated that piperine induced apoptosis and increased the percentage of cells in G_2_/M phase in 4T1 cells and induced K562 cells to differentiate into macrophages/monocytes [88, 89]. Piperine also has very good antimetastatic properties against lung metastasis induced by B16F-10 melanoma cells in mice (200 *μ*M/kg) [90] and suppresses phorbol-12-myristate-13-acetate (PMA)-induced tumor cell invasion [91].

Piperine is a potent inhibitor of NF-*κ*B, c-Fos, cAMP response element-binding (CREB), activated transcription factor 2 (ATF-2), among others. [92]. It suppresses PMA-induced MMP-9 expression via the inhibition of PKC*α*/extracellular signal-regulated kinase (ERK) 1/2 and reduction of NF-*κ*B/AP-1 activation [91]. Remarkably, piperine also inhibits the functions of P-glycoprotein (P-gp) and CYP3A4, which not only affects drug metabolism but also re-sensitizes multidrug resistant (MDR) cancer cells [93, 94]. Piperine increases the therapeutic efficacy of docetaxel in a xenograft model without inducing more adverse effects on the treated mice by inhibiting CYP3A4, one of the main metabolizing enzymes of docetaxel [95].

### 2.5. Sanguinarine

Sanguinarine (Figure 1) is a benzophenanthridine alkaloid isolated from the Papaveracea family, which includes *Sanguinaria canadensis* L. and *Chelidonium majus* L. [96, 97]. It has antibacterial, antifungal, antischistosomal, antiplatelet, and antiinflammatory properties [11, 98–100], and is used for schistosomiasis control [11]. Sanguinarine also exhibits anticancer potentials [101–104] and is currently receiving attention from researchers. Data from *in vitro* studies indicates that this alkaloid presents anticancer effects at concentrations less than ten micromoles in most cases. Sanguinarine induces cell cycle arrest at different phases or apoptosis in a variety of cancer cells [101, 102, 104–107]. It remarkably sensitizes breast cancer cells to tumor necrosis factor (TNF)-related apoptosis-inducing ligand-mediated apoptosis [105]. Sanguinarine also shows antiangiogenic effects in mice (5 mg/kg), presents anti-invasive effects, and overcomes P-gp-mediated MDR phenotype [108–110]. A strategy involving the coadministration of COX-2 inhibitors and sanguinarine has been recommended for the management of prostate cancer [111]. It has also been suggested that sanguinarine may be developed as an agent for the management of conditions elicited by ultraviolet exposure such as skin cancer [112].

The most possible mechanism responsible for the anticancer effects of this compound is its ability to directly interact with glutathione (GSH). This interaction severely depletes cellular GSH and induces ROS generation [102, 103, 105, 113]. Pretreatment of N-acetyl cysteine or catalase prevents the sanguinarine-induced ROS production and cytotoxicity [102, 113]. This mechanism is very similar to that of the TopII inhibitor salvicine, a diterpene quinone synthesized via the structural modification of a natural compound isolated from *Salvia prionitis lance *[114, 115]. Sanguinarine is a selective inhibitor of mitogen-activated protein kinase phosphatase 1 (MKP-1), which is overexpressed in many tumor cells [116]. The disruption of microtubule assembly dynamics [117], the nucleocytoplasmic trafficking of cyclin D1 and TopII [118], and the induction of DNA damage [109] also contributes to, at least in part, the anticancer effects of this compound. Sanguinarine is a potent suppressor of NF-*κ*B activation induced by TNF, interleukin-1, phorbol ester, and okadaic acid, but not that activated by hydrogen peroxide or ceramide [119]. It also effectively inhibits the signal transducer and activator of transcription 3 activation (STAT-3) [120]; downregulates CDKs, cyclins, MMP-2, and MMP-9 [107, 110]; upregulates p21, p27 [107], and the phosphorylation of p53 [101]; modulates the members of the Bcl-2 family including Bax, Bak, Bid, Bcl-2, and Bcl-xL [101, 105, 106]; activates caspases [104–106]; and upregulates death receptor 5 (DR-5) [104].

### 2.6. Tetrandrine

Tetrandrine (Figure 1), a bisbenzylisoquinoline alkaloid from the root of *Stephania tetrandra*, exhibits a broad range of pharmacological activities, including immunomodulating, antihepatofibrogenetic, antiinflammatory, antiarrhythmic, antiportal hypertension, anticancer and neuroprotective activities [11, 121]. It generally presents its anticancer effects in the micromolar concentrations. Tetrandrine induces different phases of cell cycle arrest, depends on cancer cell types [122–124], and also induces apoptosis in many human cancer cells, including leukemia, bladder, colon, hepatoma, and lung [122–130]. *In vivo* experiments have also demonstrated the potential value of tetrandrine against cancer activity [126, 127, 131]. For example, the survival of mice subcutaneously inoculated with CT-26 cells is extended after daily oral gavage of 50 mg/kg or 150 mg/kg of tetrandrine [127]. Tetrandrine also inhibits the expression of VEGF in glioma cells, has cytotoxic effect on ECV304 human umbilical vein endothelial cells, and suppresses *in vivo* angiogenesis [131]. Tetrandrine-treated mice (10 mg/kg/day) have fewer metastases than vehicle-treated mice, and no acute toxicity or obvious changes can be observed in the body weight of both groups [132].

Coadministration of tetrandrine restores the sensitivity of MDR cancer cells to doxorubicin, paclitaxel, docetaxel, and vincristine [133–135] through the inhibition of P-gp. In mice with MDR MCF-7/adr or KBv200 cell xenografts, co-administration of tetrandrine increases the anticancer activity of doxorubicin and vincristine without a significant increase in toxicity [133, 135]. Hence, tetrandrine holds a great promise as a MDR modulator for the treatment of P-gp-mediated MDR cancers. Tetrandrine appears to be a promising candidate for combining with several chemotherapeutic agents, such as 5-fluorouracil and cisplatin, *in vitro* or *in vivo* [126, 136, 137]. It enhances tamoxifen-induced antiproliferation by inhibiting phosphoinositide-dependent kinase 1 [138]. Tetrandrine also enhances the radio sensitivity of various cancer cells mainly by affecting the radiation-induced cell cycle arrest and redistributing the cell cycle [139–143]. All these observations are rational evidence supporting the application of tetrandrine as an adjunct for cancer chemotherapy or radiotherapy.

Activation of glycogen synthase kinase 3*β* (GSK-3*β*), generation of ROS, activation of p38 mitogen-activated protein kinase (p38 MAPK), and inhibition of Wnt/beta-catenin signaling might contribute to the anticancer effects of tetrandrine [126, 127, 144–146]. Tetrandrine also effectively up-regulates p53, p21, p27, and Fas [123, 124, 145, 147]; down-regulates Akt phosphorylation, CDKs, and cyclins [124, 145, 148]; modulates the members of the Bcl-2 family including Bax, Bcl-xL, and Bid [147, 148]; activates caspases [145, 147].

### 2.7. Other Alkaloids with Anticancer Effects

Aside from the aforementioned alkaloids, other alkaloids such as chelerythrine isolated from *Toddalia asiatica* (L.) Lam, chelidonine isolated from* Chelidonium majus* L., fagaronine isolated from *Fagara zanthoxyloides* Lam., lycorine isolated from *Lycoris*, nitidine chloride isolated from *Zanthoxylum nitidum *(Roxb.) DC., solanine isolated from *Solanum tuberosum*, sophocarpine isolated from *Sophora alopecuroides* L., trigonelline isolated from *trigonella foenum-graecum* also present anticancer potentials with diversiform mechanisms [11, 149–153]. However, reports on the anticancer activities and underlying mechanism of actions of these compounds are limited.

## 3. Discussion

In this paper, we summarized the recent progress of several typical alkaloids with anticancer activities and presented some characteristics of these compounds. On the basis of the previous studies, alkaloids with anticancer activities reflect diversity at least in three aspects.

**Figure 2:**
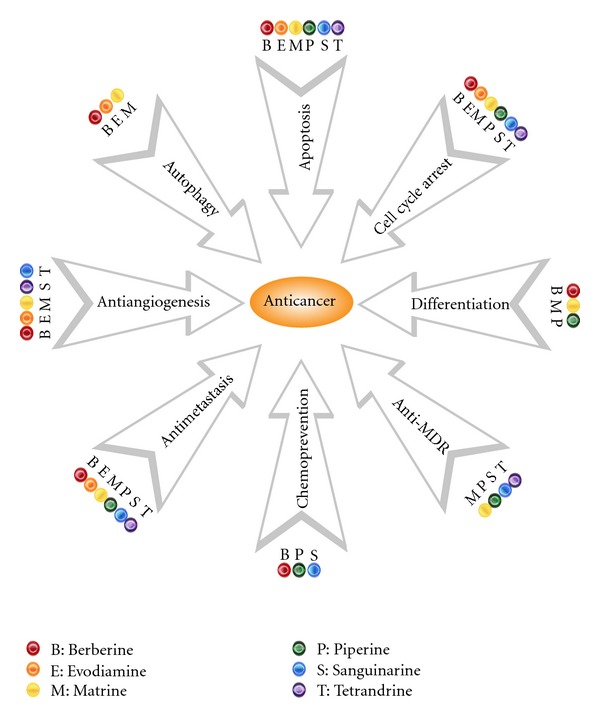
Berberine, evodiamine, matrine, piperine, sanguinarine, and tetrandrine restrain cancer by modulating multiple signaling pathways, resulting in the inhibition of the initiation of carcinogenesis, induction of cell cycle arrest, apoptosis, autophagy, or differentiation, and inhibition of metastasis, angiogenesis, and so forth.

First, the source of alkaloids with anticancer potentials is very extensive. Most of the aforementioned alkaloids are from different families, and the biosynthesis of these compounds is also varied. For example, berberine is isolated from Ranunculaceae and roots in phenylalanine and tyrosine, whereas evodiamine is isolated from Rutaceae and roots in tryptophan [1]. Second, the pharmacological activities of these alkaloids are varied [11, 12, 154]. For instance, piperine and berberine are used to treat epilepsy and diarrhea, respectively [155, 156], and both of these compounds show anticancer and other pharmacological effects. Third, the research focuses of these anticancer alkaloids are also very different. Research on piperine is usually focused on cancer prevention [82, 85], whereas that on most other alkaloids is mainly focused on cancer chemotherapy, especially on the evaluation of antiproliferative activity [12, 37, 113, 124].  Figure 2 summarizes the different roles of the aforementioned six alkaloids to achieve their anticancer effects.

In addition to their diversity, the anticancer alkaloids also have several other characteristics or/and issues which should be addressed. First, the range of alkaloid concentration necessary to elicit the anticancer effects is wide [4, 5, 12, 60, 124]. The needed concentration is relatively higher for most of the aforementioned alkaloids to produce anticancer effects, compared with the widely used chemotherapeutic drugs such as CPT [5] and vinblastine [4], although both are also naturally derived alkaloids. The concentration of matrine used to produce anticancer effects even reaches millimole [60]. Therefore, modification of the compound via chemical methods may be a good strategy. This observation also indicates that combination therapy probably provides an optimal venue for the clinical application of these compounds because most of these alkaloids exhibit synergistic or enhancement effects when combined with chemotherapeutic drugs in both *in vitro* and *in vivo* experiments [95, 136, 157, 158].

**Figure 3:**
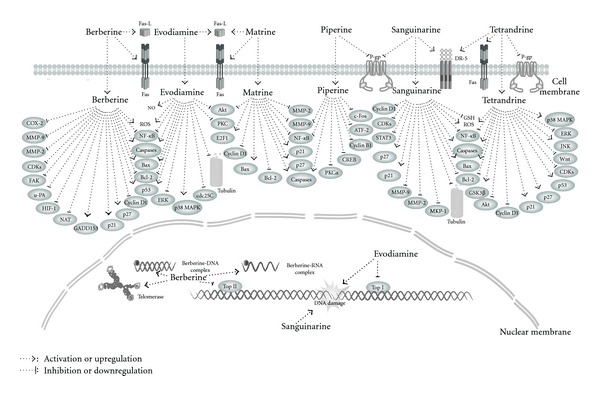
The schematic diagram of the molecular machinery and possible targets for the antineoplastic properties of berberine, evodiamine, matrine, piperine, sanguinarine, and tetrandrine. ATF-2: activated transcription factor 2; Bax: Bcl-2-associated X protein; Bcl-2: B-cell lymphoma 2; CDKs: cyclin-dependent kinases; COX-2: cyclooxygenase 2; CREB: cAMP response element-binding; DR-5: death receptor 5; ERK: extracellular signal-regulated kinase; FAK: focal adhesion kinase; Fas-L: Fas ligand; GADD153: growth arrest and DNA-damage-inducible gene 153; GSH: glutathione; GSK3*β*: glycogen synthase kinase 3*β*; HIF-1: hypoxia-inducible factor 1; MKP-1: mitogen-activated protein kinase phosphatase 1; MMP-2: matrix metalloproteinase 2; MMP-9: matrix metalloproteinase 9; NAT: N-acetyltransferase; NF-*κ*B: nuclear factor *κ*-light-chain-enhancer of activated B cells; NOS: nitric oxide synthase; p38 MAPK: p38 mitogen-activated protein kinase; PKC: protein kinase C; P-gp: P-glycoprotein; ROS: reactive oxygen species; STAT-3: signal transducer and activator of transcription 3; TopI: topoisomerase I; TopII: topoisomerase II; u-PA: urokinase-type plasminogen-activator.

Second, alkaloids isolated from natural herbs seem to have many targets to realize their multiple pharmacological effects (Figure 3), indicating that most of them are “dirty compounds.” These “dirty compounds” are a pressing medical necessity, especially for the treatment of complex diseases such as cancer [159]. However, the discovery of the molecular targets and mechanisms of these alkaloids still has a long way to go. Recent developments in biology, such as the emergence of the “-omics” fields of study, surface plasmon resonance technology, and siRNA, may greatly facilitate researches in this area [4, 160–163].

Third, most of these alkaloids have poor water solubility and low bioavailability and are hard to reach the specific cancer site. In addition to the structural modification, changing the drug delivery system could be another strategy. The development of nanotechnology may bring hope to solve these problems, and actually, there have been already some successful cases [164, 165].

Fourth, the toxicity of these compounds also cannot be ignored. For example, the most common side effects of berberine include anaphylaxis, constipation, and skin allergies [166]. Berberine can displace bilirubin from serum-binding proteins and cause kernicterus, jaundice, and brain damage in infants [166–168]. Neurotoxicity, immunotoxicity, and reproductive toxicity induced by piperine have been reported [169–171], and hepatotoxicity and embryonic toxicity can also be induced by sanguinarine [172, 173]. Therefore, alkaloids isolated from natural herbs are not always safe. The dosages, the routes of administration and the treatment procedures, among others, are very important. The transformation of chemical structures and the application of new drug delivery systems may reduce the toxicities of these compounds.

Finally, though there are several clinical studies of the alkaloids for the treatment of other diseases, for example, berberine for the treatment of diabetes or metabolic syndrome, there is no report about the clinical trial for cancer prevention or treatment using the aforementioned alkaloids. As there is a big jump from experiment researches to clinical ones, it is necessary to carry out some clinical anticancer trials for these alkaloids, such as berberine and tetrandrine.

In conclusion, for the future work in the field, (1) the exact anticancer mechanisms of alkaloids should be further identified using new pharmacological technologies; (2) the chemical structures of these lead compounds may be transformed via pharmaceutical chemistry; (3) the effective combinational therapy methods may be explored; (4) the effective drug delivery systems need to be developed; (5) the additional clinical anticancer trials for these alkaloids need to be performed.
